# Re-aligning Incentives to Address Informal Payments in Tanzania Public Health Facilities: A Discrete Choice Experiment

**DOI:** 10.34172/ijhpm.2022.6877

**Published:** 2022-12-03

**Authors:** Peter Binyaruka, Antonio Andreoni, Dina Balabanova, Martin McKee, Eleanor Hutchinson, Blake Angell

**Affiliations:** ^1^Department of Health System, Impact Evaluation and Policy, Ifakara Health Institute, Dar es Salaam, Tanzania.; ^2^Department of Economics, SOAS University of London, London, UK.; ^3^South African Research Chair in Industrial Development, University of Johannesburg, Johannesburg, South Africa.; ^4^Department of Global Health and Development, London School of Hygiene and Tropical Medicine, London, UK.; ^5^The George Institute for Global Health, University of New South Wales, Sydney, NSW, Australia.

**Keywords:** Informal Payment, Preferences, Policy Options, Discrete Choice Experiment, Tanzania

## Abstract

**Background:** Informal payments for healthcare are typically regressive and limit access to quality healthcare while increasing risk of catastrophic health expenditure, especially in developing countries. Different responses have been proposed, but little is known about how they influence the incentives driving this behaviour. We therefore identified providers’ preferences for policy interventions to overcome informal payments in Tanzania.

**Methods:** We undertook a discrete choice experiment (DCE) to elicit preferences over various policy options with 432 health providers in 42 public health facilities in Pwani and Dar es Salaam region. DCE attributes were derived from a multi-stage process including a literature review, qualitative interviews with key informants, a workshop with health stakeholders, expert opinions, and a pilot test. Each respondent received 12 unlabelled choice sets describing two hypothetical job-settings that varied across 6-attributes: mode of payment, supervision at facility, opportunity for private practice, awareness and monitoring, measures against informal payments, and incentive payments to encourage noninfraction. Mixed multinomial logit (MMNL) models were used for estimation.

**Results:** All attributes, apart from supervision at facility, significantly influenced providers’ choices (*P*<.001). Health providers strongly and significantly preferred incentive payments for non-infraction and opportunities for private practice, but significantly disliked disciplinary measures at district level. Preferences varied across the sample, although all groups significantly preferred the opportunity to practice privately and cashless payment. Disciplinary measures at district level were significantly disliked by unit in-charges, those who never engaged in informal payments, and who were not absent from work for official trip. 10% salary top-up were preferred incentive by all, except those who engaged in informal payments and absent from work for official trip.

**Conclusion**: Better working conditions, with improved earnings and career paths, were strongly preferred by all, different respondents groups had distinct preferences according to their characteristics, suggesting the need for adoption of tailored packages of interventions.

## Background

**Key Messages**

** Implications for policy makers**

- Top-down or ‘traditional’ anti-corruption measures, such as transparency, accountability, and sanctions, are important but not sufficient, while bottom-up measures based on what providers perceive as important are more likely to be acceptable.
- Supporting healthcare providers with interventions such as offering opportunities for private practice and rewarding/incentivising adherence to rules may be a more effective use of scarce resources to achieve reductions in informal payments than greater monitoring and punishment of offenders.
- Policies and programmes should also seek to improve working conditions by improving remuneration and moving away from cash transactions as highly acceptable interventions to reduce the chances for engaging in informal payments.

** Implications for the public**

Informal payments compromise delivery of healthcare and the public can reasonably expect that authorities will prevent them. Health providers are amenable to measures that would do this but the package of measures should be adapted to the characteristics and preferences of the work force. Attempts to reduce informal payments should begin by identifying what strategies are preferred most by health providers, to design bottom-up approaches to maximize their acceptability. Offering incentives for good behavior such as opportunity for private practice, better working environment, better remuneration, and career growth opportunities are highly acceptable to health workers and strongly preferred over measures that threaten punishment for breaches.

Informal payments are common in health systems in many low- and middle-income countries. They include payments for care or for health supplies that are formally covered by the health system, made to individuals or institutional facilities, in kind or in cash, and are unregulated or illicit.^1,2^ They are a consequence of the power imbalance between health workers and patients^3-5^ in situations where public health facilities are underfunded and the rewards to health workers fail to meet their expectations given their personal investment in training.^6^ Like other out-of-pocket (OOP) costs, they are inequitable and inefficient, disproportionately impacting vulnerable groups and are a major barrier to achievement of universal health coverage.^7-9^ Yet they remain an intractable problem and we know little about what might work to reduce them.

 Traditionally, anticorruption research in the health sector has been dominated by frameworks that see rule-breaking (including the levying of informal payments) as a consequence of poor governance.^10^ From this viewpoint, interventions developed to overcome informal payments have focused on improving transparency and accountability through regulatory and enforcement mechanisms. Examples include creation of an independent authority to investigate and punish corruption, increasing awareness of the unacceptability of these practices among health workers and the public, paying health workers more, and enforcing disciplinary measures.^11^ There is little evidence that these strategies work, in part because they fail to consider the political economy in which they take place. In particular, limited attention has been given to the ways in which various incentives affect health workers and institutions differently, and how each respond to them.^6,12,13^

 Recent political economy frameworks developed to investigate rule-breaking in the public sector may offer a means to address this issue.^14,15^ By recasting them as a structural rather than moral issue, recognising broader systemic factors acting influencing this behaviour, effective interventions may be possible. The Anti-Corruption Evidence (ACE) research programme,^13^ undertaken in Tanzania, Nigeria and Bangladesh, uses a political settlement framework to understand behaviour of actors in the health system.^14,16^ It focuses on informal practices and processes that create vulnerability to corruption at the sectoral level. The ACE approach points to two key issues, that formal rules are often weakly enforced and that they are widely violated by powerful agents. In Tanzania’s political settlement these two challenges have adversely affected development in several sectors, but in different ways.^17^ Consequently, even in the same country it is necessary to understand how the distribution of power – its political settlement – and incentive structures arising from opportunities for rent-capture and vulnerability to corruption, apply in each sector.

 The qualitative research that informed this analysis confirmed a perception that informal payments are widespread throughout the Tanzanian health system, particularly in urban hospitals and health centres.^18^ They included payments to bypass long queues, staff sharing per diems for seminars, preferential treatment, gifts in appreciation of services provided, and selling medical commodities that should be provided for free. The facilitators of informal payment included low salaries, shortage of health workers, and lack of timely payment of entitlements, medical equipment, supplies, and supervision. There were also socio-cultural facilitators, such as perceptions of what is expected. The propensity to engage in informal payments varied among departments (more common in labour and delivery wards), at different times and days (common at night and weekends when managers were absent), and among different cadres. Health providers tend to work together to obtain informal payments, for example with nurses often organising them and sharing with doctors.

 In a setting where informal payments are widespread, top-down anti-corruption strategies and measures to promote transparency are only likely to work if they are supported by bottom-up strategies that consider the incentives of the actors involved and how power is distributed among them. We will only be able to design anti-corruption strategies that are feasible and effective if we have granular evidence on the various incentives driving the behaviour of health workers and how those with different amounts of power within a health facility can be encouraged to adopt more desirable practices. By these means we can reduce the facilitators of informal payments through bottom-up measures that align with their individual, and potentially differing, incentives and preferences. This approach has only recently been discussed in the health sector and there is scarce empirical evidence about how to design jobs that reduce the scope for informal payments.^10^ In other sectors, such supportive policy settings have led to new approaches to enforcement where the rule-following majority engages in peer-monitoring.^13^

 In this paper we sought to narrow this knowledge gap using a discrete choice experiment (DCE) survey in Tanzania. DCEs measure stated preferences by asking respondents to make a series of choices between a number of hypothetical alternatives that differ across several key factors.^19-21^ The DCE was preferred as a means to identify the bottom-up strategies by eliciting providers’ preferences for policy interventions to overcome informal payments. This is, to our knowledge, the first DCE undertaken for this purpose although this method has been used widely for other purposes in the health sector, including in low- and middle-income countries, to investigate patient, policy-maker and health worker preferences over policy options or different job attributes^20,22-24^ and different payment methods.^25^ The preliminary findings of this study, reporting initial analyses prior to full development of the model, were presented at the 16th World Congress of Public Health, held virtually, in 2020.^26^

## Methods

 This DCE was part of a mixed-methods study to investigate the determinants and operation of informal payments in the Tanzanian health system and develop feasible strategies to overcome them.

###  Study Context

 This study was conducted in Tanzania, a lower middle-income country in East Africa, with an estimated population of around 60 million people in 2022.^27^ Healthcare provision is dominated by public facilities (70%), with other services provided by private and faith-based organizations.^28^ The Tanzanian health system is beset by systemic challenges. These include poor working environments (eg, shortages of drugs and supplies, poor housing for staff, inadequate salaries, limited and unpredictable allowances), shortage and maldistribution of healthcare workers, and inadequate funding to cover operational costs.^29-31^ Healthcare in Tanzania is financed from multiple sources: in 2015/2016, about 34% was from tax revenue, 36% from donor support, 8% from health insurance and 22% through OOP payments.^32^ About 32% of Tanzanians were covered by different forms of health insurance in 2018,^31^ leaving the majority uninsured, especially those in rural areas, the poor, and those working in the informal sector. Although the poorest and vulnerable groups (eg, pregnant women, children, and elders) are exempted from direct payments, this policy is poorly enforced and they often still pay OOP, both formal and informal.^33-36^ While the true extent of informal payments is unknown, they have been shown to be common across the Tanzanian health system.^37,38^

###  Hypotheses

 Following from our political economy framework outlined above, we tested three main hypotheses:

- Incentives and rewards that involve bottom-up peer-monitoring will be highly preferred by health workers. This will encourage health workers to follow the rules but will differentially influence the behaviour of different groups.
- Formalising existing informal practices, for example by allowing time off for private practice and other commitments, will be highly valued by health workers. This may address shortcomings in the system that drive some providers to break rules out of necessity. In doing so, they may create conditions where rule-breaking is undertaken by an increasingly small subset of providers, reducing the incentive for others to levy informal payments.
- Traditional top-down measures such as oversight and supervision and the imposition of punishment, transparency and accountability measures, such as providing information (eg, on a noticeboard) to the public, will be acceptable to providers as typically done for other top-down measures. However, the effectiveness of these measures might not be realised unless accompanied by measures to address underlying shortcomings in the system that drive rule-breaking but may not significantly influence the incentives facing providers in situations where they have the power to circumvent them.

###  DCE Setting and Instrument Development

 As recommended in the DCE literature, we followed a multi-stage, mixed-methods approach to identify attributes and levels relevant to our research hypotheses.^39^ The development of DCE attributes and levels involved five main stages: a scoping literature review,^38^ qualitative data collection, a workshop with health providers and managers, expert opinions to narrow and fine-tune the context-specific attributes, and a DCE pilot study. First, the scoping review examined corrupt practices in the Tanzanian health sector, identifying informal payments as a very common and particularly harmful form of corruption. It identified potential facilitators (ie, individual and systemic factors) and responses (ie, social accountability, performance-based financing, and enhancing insurance coverage). Second, 27 in-depth qualitative interviews with frontline public health workers from 9 facilities and health managers at the district level were conducted to further identify individual and systemic facilitators and potential responses including effective round the clock supervision, improving the working environment and entailments, and promoting public awareness of entitlement to services. Third, a series of candidate attributes were derived and presented at a consensus-building workshop with 10 health providers from 5 facilities and 8 managers to validate and agree on the most important facilitators/drivers of informal payments and potential areas for policy intervention. Fourth, the potential DCE attributes and levels were reviewed by a panel of multidisciplinary experts (PB, AA, DB, MM, EH, BA) with expertise in public health, health policy, economics, and health systems research in order to fine-tune the attributes and levels. Lastly, we piloted the proposed attributes and levels with 15 health providers from 9 public health facilities. The pilot study found the wording and numbers of the attributes and levels were acceptable to participants, all questions were understandable, and respondents appeared to be trading off the different attributes/levels. After going through all these stages, we identified the following six job attributes (Table 1): *mode of payment, supervision at the facility level, opportunity for private practice, awareness and monitoring, measures against informal payment, and incentive payment for lack informal payment in the past 6 months*.

**Table 1:** Discrete Choice Experiment Attributes and Levels for Health Providers in Tanzania

**Attributes**	**Levels**	**Hypothesis/Rationale/Proposition **
Mode of payment	Cashless payment (electronic/insurance payment only) Cash only (base category)	These were expected to differentially impact providers based on their position in the facility with more senior staff able to bypass these measures.
Supervision at facility	Rotating supervisor at facility present 24 h/d Supervisor at facility 7:30 am-4 pm on weekdays (base category)	Providers expected to avoid informal payments in the presence of supervision. However, supervision may also help in clinical guidance and support in the facilities.
Opportunity for private practice	Dedicated time off each week (including agreement for private practice) None (base category)	Providers expected to prefer jobs that formalise an opportunity to earn income through private practice.
Awareness and monitoring	Receipts required for all transactions Facility noticeboard displaying services provided and correct fees Hotline to anonymously report informal payment to health manager/board None (base category)	Providers expected to be amenable to community monitoring and transparency mechanisms if accompanied by a reduction in formal monitoring.
Measures to address informal payment	Preferential training/promotion for providers in facilities with no infraction for past year Disciplined at district level (eg, warning letter that reduces opportunity for promotion) Disciplined within facility (eg, official warning) (base category)	Providers expected to dislike higher-level disciplinary action but be receptive to positive rewards for good performance.
Incentive payment for staff no informal payments are recoded at the facility in past 6 months	10% of base salary 5% of base salary No incentive payment on top of regular salary (base category)	Health providers expected to prefer jobs with incentive payments, even if these are conditional on monitoring of no informal payments.

 We generated the choice sets/scenarios based on the number of attributes and levels. Our study has three attributes with two levels, two attributes with three levels, and one attribute with four levels (Table 1). Through a *full factorial design*, one can generate 288 possible job descriptions (2^3^ x 3^2^ x 4^1^) with various combinations of levels from six job attributes. Since those number of scenarios are unmanageable to administer, we used *fractional factorial design*^39^ to reduce the number of choice sets from 288 to 12. We used a D-efficient DCE design with 12 scenarios using NGENE software (version 1.2.1). The final DCE tool included 12 choice sets with respondents asked to select between two job sets (Job A and Job B) or neither job as an opt-out option (Figure shows one of the 12 choice sets). We included an opt-out option in line with recommendations in the literature since forcing providers to make a choice on job alternatives only can lead to over-estimation of utility for parameters.^40^ The DCE included the following introductory statement for respondents: *Imagine that you are considering two hypothetical offers for jobs at different facilities. You are given a choice between Job A and Job B. Both jobs are the same in terms of the duties, designation, job title and benefits apart from the ways shown here. For each question, please choose whether you would accept Job A, Job B, or neither of them if they were offered to you. If you select neither we would still like to know whether you think Job A or Job B is better.*

**Figure:**
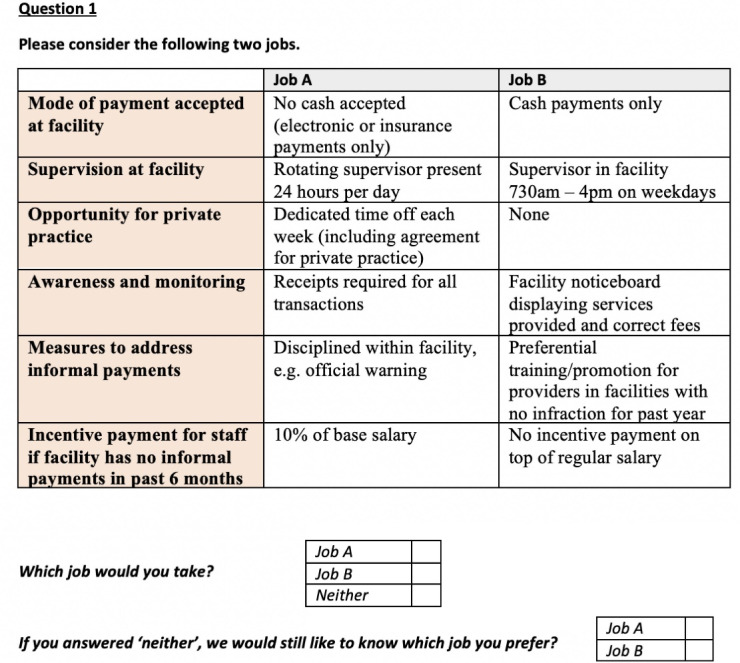
Example of Choice Set.

###  Data Collection

 Data were collected in a cross-sectional survey of health workers in six districts in Pwani region and five districts in Dar es Salaam region, Tanzania, incorporating urban, peri-urban and rural settings. The survey was undertaken from July to August 2019 in 42 health facilities. We included all public hospitals (n = 14, 33.3%) and health centres (n = 28, 66.6%) in two regions, excluding only national, military and specialised hospitals. Calculating a sample size for DCE studies is a contested issue in the literature.^23,41-43^ Since we planned to investigate heterogeneity/subgroup analysis across our sample, we aimed for a sample at least 400 health workers to ensure a large enough sample to investigate differences between subgroups in line with similar studies in the literature.^44^ We drew a convenience sample of health workers from those present at surveyed facilities on the day, selected purposively to include individuals in different departments and levels of seniority. We included a minimum of five medical staff members at each facility. The final sample comprised 432 health workers from all 42 facilities (Table 2). 188 (43.5%) worked in hospitals and 244 (56.5%) in health centres.

**Table 2:** Sample Characteristics

	**Number of Healthcare Workers**	**%**
Region	(n = 432)	
Dar es Salaam	194	44.9
Pwani	238	55.1
Facility level of care	(n = 432)	
Hospitals	188	43.5
Health centres	244	56.5

 The data collection tool captured data on the respondents’ socioeconomic and demographic characteristics as well as facility level characteristics. It was translated from English to Swahili and programmed digitally into tablet devices for easy data collection, with a paper-based questionnaire made available for quick reference by enumerators and respondents. All interviews for data collection were done in Swahili between July and August 2019, and a single interview took around one hour on average. Field enumerators underwent training on the survey and DCE method before the pilot study and actual data collection. The pilot indicated that respondents had a clear understanding of the choice exercise and were able to trade-off between levels.

###  Data Analysis

 The analysis used discrete choice models. The choice model analyses are based on random utility theory which assumes that the utilities that respondents attach to any of the alternative jobs are determined by their attributes and attribute-levels.^45^ This implies that decision-maker *n* is assumed to be a rational decision-maker facing a choice among *J* alternative jobs with various attributes and levels. The decision-maker is assumed to choose an alternative with the highest level of utility such that they will choose alternative job *i* over alternative job *j* if and only if the utility of job *i* is greater than utility of job *j*, ie, *U*_in_* ≥ U*_jn_ for *i ≠ j.* Since the utility of the decision-maker is not directly observable, the utility of choosing an alternative job *i* is expressed as linear function of observed characteristics plus an error term^46,47^:

*U*_in_= *V*_in_+*ε*_in_

 The researcher can observe *V*_in_ which includes observable characteristics, while *ε*_in_ is a random component and unobservable. Hence, the utility derived from the choice of alternative job *i* is expressed as a functional of job attributes plus a random component. Let *U*_in_ denote the utility derived by individual consumer *n* through choosing alternative job *i*:

*U*_in_= *β*_i_*X*_in_+ *ε*_in_

 Where, *β*_i_ is the observed vector of parameters for attributes with respect to alternative *i, X*_in_ is the observed vector of job attributes influencing the choice of alternative job *i* in order to derive utility, *ε*_in_ is the random error term reflecting random choice behaviour or unobserved factors, and *β*_i_*X*_in_ is a matrix of job attributes which reflects the deterministic or observed portion of the utility. The coefficients of interest are *β*_i_’s as they provide quantitative information on the strength of preference for each attribute level, as well as trade-offs, monetary values, and predicted take-up of alternative. The positive (negative) *β*_i_ coefficient indicates an individual’s utility (disutility) from the use of the chosen job attribute.

 By assuming the random error component *ε*_in_ is independent and identically distributed (IID) across alternatives and respondents, then the probability of choosing job *i* from J alternative jobs can be expressed in a conditional logit formula below^45,47^:


$$P_{in}=\frac{e^{\beta_{i}X_{in}}}{\sum j\;e^{\beta_{i}X_{jn}}}$$

Thus, our main effects and subgroup effects were estimated through a mixed multinomial logit (MMNL) model, because conditional logit model works only if the IID assumption is true, and does not allow the assessment of preference heterogeneity across respondents.^48^ The MMNL model relaxes the IID assumption.^49^ The output of a MMNL model includes mean coefficients (*β*_i_ representing the relative utility of each attribute conditional on other attributes), and standard deviations of the random coefficients (reflecting the degree of heterogeneity among respondents), as well as their respective *P* values and confidence intervals. All attributes were effects coded and the attribute for financial incentive/salary top-up was analysed as both a continuous and categorical variable, with the aim of testing the presence of any non-linear effect of categorical incentive. The distribution of all variables was assumed random with normal distribution.

 We also computed the relative importance for each attribute, which reflects how providers valued different attributes. We specifically used the contribution to the overall model log likelihood through partial log likelihood estimation procedure.^50^ The relative importance of each attribute was presented as percentage share and ranked in terms of order of importance. Sub-group analyses were conducted to capture preference heterogeneity across various providers’ subgroups.^39,51^ The sub-group analyses used three groups in our sample: (1) those who reported previously engaging in informal payment (27%); (2) staff in-charge of their units/departments (28%); and, (3) those who had missed work over the past month for official reasons (14%) as our qualitative research suggested that trips to seminars or training were often used as rewards by groups working together to levy informal payments. These groups were chosen due to their particular policy importance in engaging in and overcoming informal payment in Tanzania. All analyses were conducted in STATA version 16 and NLOGIT version 6.0.

## Results

###  Descriptive Statistics

 Out of 432 health workers in our sample, the majority were female, nurses and midwives, married, not in-charges of units, and relied predominantly on their primary job as their main source of income (Table 3). The average years of experience at their working station was around 7 years. In terms of facility characteristics, a few facilities had supervision throughout (40%) and electronic modes of payment (26%). Most facilities had an accountant (91%), noticeboard for displaying procedures and regulations (93%), and health facility governing committee (HFGC) (58%) (Table 3). The average perceived rating (on a scale of 1 to 10) of working environment condition was 6.6 for availability of medical commodities and facility infrastructure condition; while staffing level and provision of entailment and benefits were rated around 4.3 (Table 3).

**Table 3:** Health Workers’ and Facility-Level Characteristics

**Variables**	**Description**	**Sample (n = 432)**	
		**No.**	**%**
Individual Level Factors			
Age groups (y)	20–34	154	35.7%
	35–44	135	31.3%
	45–60	143	33.1%
Medical cadres	Medical specialist	31	7.2%
	Medical officer and clinical officer	117	27.1%
	Nurse and midwives	167	38.7%
	Others (eg, paramedics)	117	27.1%
Gender	Female	275	63.7%
Marital status	Married	306	70.8%
Position at the facility level	In-charge of the department/unit	199	46.1%
Experience at the facility level	Number of years at a facility [SD]	432	6.9 [7.5]
Supplementary job for income	Has any supplementary job	168	38.9%
Facility level factors			
Supervision throughout	Facility with supervision throughout	171	39.6%
Electronic mode of payment	Facility with electronic payment	114	26.4%
Availability of an accountant	Facility with an accountant	393	91.0%
Availability of a noticeboard	Facility with a noticeboard	403	93.3%
Availability of HFGC	Facility with a HFGC	249	57.6%
Availability of health commodities	Average rating between 1–10 [SD]	432	6.6 [2.2]
Facility infrastructure condition	Average rating between 1–10 [SD]	432	6.7 [2.3]
Staffing level	Average rating between 1–10 [SD]	432	4.5 [2.1]
Entitlements & benefits condition	Average rating between 1–10 [SD]	432	4.3 [2.5]
Facility level of care	Hospital	188	43.5%

Abbreviations: SD, standard deviation; HFGC, Health Facility Governing Committee. Mean age was 40.2 years (SD = 9.7).

###  Main Effects of the Model 

 We estimated two unforced MMNL models with continuous and categorical salary top-up. The log likelihood ratio test of the two models showed a *P* value of 1, indicating insignificant difference between models in terms of explaining variations in the outcome. Our sample of 432 respondents generated 5,184 valid responses, with only 107 responses (2.1%) were for the opt-out/status-quo scenario. Thus, we only present results for unforced model since there were few respondents who opted out. The discussion of our results focuses on model 1 in Table 4 (unforced with categorical salary top-up), since the model with a continuous incentive obscured the non-linear effect of categorical incentive (ie, on average, respondents preferred only incentives of 10% and were not significantly affected by the presence of a 5% incentive).

**Table 4:** Main Effects From Mixed Multinomial Logit Estimation

**Job Attributes/Level**s	**Unforced MMNL**			
	**Model 1: Categorical Incentive**		**Model 2: Continuous Incentive**	
	**Mean ** ***β***_i_	**Estimated SD for Random Parameters**	**Mean ** ***β***_i_	**Estimated SD for Random Parameters**
	**(1)**	**(2)**	**(3)**	**(4)**
Mode of payment				
Cashless (eg, electronic payment)	0.174***	0.374***	0.178***	0.383***
Cash only (reference)				
Supervision at the facility level				
Throughout oversight 24 x 7	0.015	0.245***	0.015	0.239***
Supervision weekdays only (7:30 am - 4 pm) (reference)				
Opportunity for private practice				
Dedicated time-off for dual practice	0.245***	0.305***	0.246***	0.304***
None (reference)				
Awareness and monitoring				
Receipt provision	0.121***	0.182**	0.119***	0.147*
Noticeboard availability	0.177***	0.119	0.176***	0.097
Hotline for reporting	0.021	0.107	0.023	0.162**
None (reference)				
Measures to address informal payment				
Promotion/training if no infraction	0.109***	0.057	0.106***	0.155***
Disciplined at district level	–0.088***	0.149**	–0.093***	0.064
Disciplined at facility level (reference)				
Incentive payment as salary top-up (per 1% increase)			0.077***	0.059***
10% incentive	0.293***	0.306***		
5% incentive	0.185	0.049		
No incentive (reference)				
Opt-out (constant)	–4.508***	1.944***	–4.326***	2.124***
Number of respondents	432		432	
Log likelihood	–3673.194		–3675.578	
McFadden pseudo R^2^	0.3550375		0.3546189	
AIC	7390.4		7391.2	

Abbreviations: SD, standard deviation; MMNL, mixed multinomial logit; AIC, Akaike Information criterion. *** denotes significance at 1%, ** at 5%, and * at 10% level.

 The result of the main effects showed that all attributes, apart from supervision at facility, significantly influenced health providers’ choices of job type (Table 4). All parameter estimates had the expected positive signs indicating they were liked by respondents, except for a negative sign of disciplinary measures at the district level. In particular, incentive payment as 10% salary top-up and opportunity for private practice were the major predictors in providers’ choices of job type by having relatively larger positive coefficients (*β* = 0.293, *P*< .001) and (β = 0.245, *P*< .001), respectively. Less preference for disciplinary measures at district level was the only significant predictor that influence negatively the job choice, but with a relatively small negative coefficient (*β* = -0.088, *P*< .001) (Table 4).

 More specifically, on average, health providers preferred cashless modes of payment rather than cash transactions, and had a strong preference for jobs offering an opportunity for private practice (Table 4). Providers significantly preferred a job at a facility with noticeboards and which required receipts, both acting as awareness raising measures, but the existence of a hotline for reporting infractions did not significantly influence the choices of respondents. Providers preferred a reward in terms of promotion/training opportunity if a facility does not have informal payments for a past year and disliked jobs where disciplinary measures were handled at the district rather than facility level, however, the coefficients for these were smaller than that for other attributes. As expected, we found a positive coefficient for incentive payment/salary top-up, which indicated that health providers’ preference for a job increases as salary top-up increases, however, this was only present for a 10% incentive; a 5% incentive was not significantly associated with respondent choice. The lack of linearity in incentive payments also limited the analysis of willingness to sacrifice. The constant or opt-out term was negative and statistically significant indicating that providers strongly preferred accepting one of the jobs presented compared to opting out/remaining with a status quo job. The estimated significant standard deviations suggest substantial heterogeneity in providers’ preferences for all attributes (Table 4).

###  Relative Importance of the Attributes

 The top preferred attribute with greatest impact on job choice was incremental incentive/salary top up, followed by dedicated time-off for private practice and cashless mode of payments (Table 5). These three attributes had a cumulative share of 85.6% in influencing respondents’ job preferences. However, incentive payment and opportunity for private practice alone accounts for 60.2%. The next attributes in the order of importance were awareness and monitoring measures, and supervision at facility, while measures against informal payments (mainly promotion/training as rewards than insignificant disciplinary measures at district level) had very least contribution to overall preferences.

**Table 5:** Partial Log-Likelihood Analysis of Ranking Relative Importance of Attributes

**Attribute**	**Log Likelihood**	**Partial Effect**	**Relative Effect (% of Total Change)**	**Cumulative (%)**	**Order of Impact**
None (full model)	-3673.194				
Incentive payment	-3826.719	-153.525	0.340	0.340	1
Opportunity for private practice	-3791.814	-118.620	0.262	0.602	2
Cashless mode of payment	-3788.027	-114.832	0.254	0.856	3
Awareness and monitoring	-3709.256	-36.062	0.080	0.936	4
Supervision at facility	-3691.157	-17.963	0.040	0.975	5
Measures to address informal payment	-3684.307	-11.113	0.025	1.000	6

###  Subgroup Effects

 Generally, all groups significantly preferred the opportunity to practice privately and cashless payment (Table 6). However, based on the magnitude of the coefficients, the respondents with managerial roles (in-charge of units) had a much stronger preference for jobs with opportunities for private practice, potential promotion or training benefits for good performance and 10% incentive payments than other respondents. They significantly disliked stronger punishment at the district level, which did not significantly influence the choices of staff with no managerial roles.

 Respondents who reported previously engaging in informal payment had a much higher preference than others for accepting a job. They were not significantly influenced by traditional approaches to overcoming informal payments including additional financial incentives, having supervision throughout a facility or the stronger discipline at the district rather than facility level. They had a strong preference for jobs affording them the opportunity for private practice and a smaller but significant preference for jobs where good performance was rewarded with promotion or training opportunities and were open to interventions to improve public oversight preferring jobs with cashless payment and a public noticeboard. There were no attributes that this group significantly disliked.

**Table 6:** Sub-group Analysis

**Job Attributes**	**Staff Position**		**Ever Engaged in Informal Payment Before **		**Been Absent for Official Reasons**	
	**Unit In-charge**	**Normal Staff**	**Yes**	**No**	**Yes**	**No**
	**n = 119**	**n = 233**	**n = 117**	**n = 315**	**n = 62**	**n = 370**
Cashless mode of payment	0.319***	0.107***	0.234***	0.161***	0.178**	0.175***
Supervision throughout at the facility	0.059*	-0.014	0.06963	0.002	0.028	0.018
Opportunity for private practice	0.321***	0.198***	0.321***	0.219***	0.289***	0.234***
Monitoring and awareness						
Receipt provision	0.141**	0.097*	0.095	0.124***	0.035	0.134***
Availability of a noticeboard	0.140**	0.211***	0.175**	0.178***	0.103	0.192***
Availability of a hotline for reporting	0.114*	-0.050	-0.048	0.039	-0.019	0.017
Measures against informal payment						
Disciplinary measures at district level	-0.141***	-0.053	-0.068	-0.101***	0.083	-0.110***
Promotion/training opportunity	0.180***	0.076**	0.126**	0.100***	0.056	0.106***
Incentive payment as salary top-up						
5% incentive	0.140	0.227	0.387	0.171	0.471	0.123
10% incentive	0.402***	0.237**	0.169	0.312***	0.109	0.338***
Opt-out	–4.375***	-4.643***	-7.220***	-4.213***	-5.295***	-4.134***
Log likelihood	–1610.373	-2039.511	-933.192	-2716.488	-510.852	-3147.027
McFadden pseudo R^2^	0.3861704	0.3360361	0.3949941	0.3458587	0.3750022	0.3548315
AIC	3264.7	4123.0	1910.4	5477.0	1065.7	6338.1

Abbreviation; AIC, Akaike Information criterion. Notes: *** denotes significance at 1%, ** at 5%, and * at 10% level.

 The group that had previously been absent for official reasons also had a strong preference for accepting a job but their decisions were only significantly influenced by the opportunity for private practice and to work at a facility with cashless payment.

## Discussion

 We estimated preferences for job attributes among public health providers in two regions in Tanzania to assess their response to potential policy interventions to address informal payment, a systemic challenge across the Tanzanian health system. To our knowledge, this is the first study using DCE to examine health workers’ preferences over policy interventions to overcome informal payments in the health sector. While interventions to improve working conditions were generally popular, potential interventions were shown to impact on respondent choices differently within the sample, with different groups of workers responding differently to the attributes presented. This finding is important for informing the development of a package of feasible interventions to overcome informal payments and challenges the applicability of traditional frameworks that have tended to view such interventions as applicable to a homogenous set of rule-breakers. Our results suggest that targeted interventions to address the underlying facilitators of behaviours may help to reduce the level of informal payment incrementally across the health system.

 The findings in this study generally support our three hypotheses, with the exception of the third that was only partially supported, with mixed findings. For instance, our findings on incentives and rewards are consistent with our first hypothesis. We expected attributes associated with higher incomes (salary top-up and private practice) or helping future promotion to be highly valued by health workers, hypothesising that such interventions could balance the presence of negative interventions such as increased monitoring or punishment. This was borne out in our data. Crucially though, simply improving salaries without understanding the diversity of staff may lead to excessively high levels of incentives (and implementation costs) that fail to affect behaviour. Respondents were on average unaffected by an incentive payment worth 5% of their base salary, only altering their choices for 10%, suggesting there is likely a minimum threshold to alter behaviour. Further, those who had previously reported engaging in informal payment were not significantly influenced by the presence of the incentive payment, casting doubt on the potential to feasibly utilise incentive payments to rid the system of informal payments and highlighting that improving incentives without understanding doctor heterogeneity may lead to high-cost interventions that still fail to work.

 With regard to our second hypothesis of formalising informal practices, the ability to practice privately was consistently highly valued by all groups. This is one element in a strategy to improve conditions and behaviour of health workers, formalising what in many situations is already existing practice. This is in line with findings previously reported in other settings.^52,53^ This attribute appealed to all three subgroups – with those in charge of units, those who reported previously engaging in informal payments and those who reported being absent from work in the past month due to official trips – by having a strong preference. Indeed, the group who had been absent for official trip shows it as the only significant attribute alongside cashless payments. Previous work has highlighted the importance of context with private practice. A review by Ferrinho, Van Lerberghe, Fronteira, Hipolito, Biscaia^54^ found that the consequences of dual practice varied by location (more in urban), medical cadre (more among educated) and ongoing reforms. Our setting, for instance, was largely urban/peri-urban where private facilities are relatively common, which may partly explain the higher preference for private practice. However, allowing dedicated time off for private practice would affect service quality so it needs carefully consideration before recommending it.

 Our findings were mixed regarding the third hypothesis about traditional top-down measures, since not all of them were preferred as expected. In particular, health workers significantly preferred promotion a reward for non-infraction, and disliked disciplinary measures imposed at the district level in response to infractions. They also preferred cashless payment and awareness/monitoring measures (ie, receipt provision and presence of noticeboard). However, the evidence on the effectiveness of these top-down measures of governance and accountability are lacking.^6,10,11^ In this regard, we argue that the effectiveness of these measures might not be realised and/or may not significantly influence the incentives facing providers in situations where they have the power to circumvent them, unless accompanied by measures to address underlying shortcomings in the system that drive rule-breaking.

 In understanding the role of financial incentives as our most preferred attribute, context matters ^55^; for instance, staff may seek an alternative to supplement their earnings (eg, seeking informal payments) when government funding is unreliable.^56^ Also, poor remuneration may demotivate staff and potentially reinforce rent seeking behaviour (survival corruption) as a coping mechanism.^6^ This is supported by an earlier analysis in this study, where we found provision of entitlements and benefits reduced taking informal payments.^37^ Several studies have also identified low income as an important determinant of informal payment.^57-62^ Combined with our results, these show that staff are currently working in an environment characterised by multiple problems and improvements in conditions may incentivise improved performance in a wide range of areas.

 The finding of less preference for disciplinary action at the district rather than facility level is likely representing discomfort with involvement of outsiders and disruption of existing networks within facilities that tolerate informal payments. This, however, varied across providers’ sub-sample. Those in charge of their units, who formally derive much of their power from within the system, had a strong negative preference for involving higher-level authorities. On the other hand, those who reported having previously engaged in informal payment and those who reported being absent from work in the past month on an official trip were not significantly influenced by the presence of the punishment attribute, potentially suggesting they have developed workarounds to these processes. Similarly, the presence of 24-hour supervision at the facility was not significantly associated with the choices of respondents in the entire sample or any subgroup, suggesting that it may not be helpful. The qualitative research that informed the DCE found that facilities have supervisors but are not around throughout as they often absent at nights and weekend, and informal payments are managed by networks of staff who may collude with facility supervisors.^18^

 The finding of higher preference for jobs with opportunities for career development through promotion or training opportunities for those who complied with the rules is in line with literature on health worker motivation and retention in Tanzania^44,63^ and elsewhere.^24,52,64^ However, in our study the preference for this attribute was relatively small (in contrast to the literature), potentially a result of being tied to the group incentive of working in a facility with no infractions (in contrast to the ability to practice privately that was not tied to any performance indicator). Some health providers might have less preference for the opportunity for career development given that it is tied to infractions reported at the whole facility (group incentive), since they may be concerned about being impacted by the behavior of others that they cannot control. It is possible that many staff do not see their careers progressing in the public sector through training and instead having a strong preference to move to the private sector.

 While participants were generally against monitoring from above, they were open to more localised interventions such as the provision of noticeboards, receipts, and cashless payments. These are important top-down measures for transparency and accountability, but are possibly preferred as visible strategies that allow to signal commitment to following the rules in a facility and obscure actual informal practices. On the other hand, these are also preferred because they can easily be bypassed by ‘powerful’ staff – for example specialists working in high demand areas and providers with wider networks. This is supported with our prior analysis as we found insignificant influence of monitoring and awareness in reducing informal payments,^37^ hence suggesting a combination of acceptable interventions (top-down and bottom-up) in an attempt to reduce informal payments. The finding of relatively strong preference for cashless payment can be explained with the possibility of respondents equating cashless payment with being able to work at a more modern, technologically advanced facility and thus preferred these jobs. The fact that some of these measures are newly implemented across facilities (eg, noticeboards, cashless payment), this perhaps might have influenced providers in choosing a job type with these developments without considering the associated implications for accountability, transparency and monitoring.

 Our findings have important policy implications. The significant preferences for all job attributes, except supervision, suggest that policy-makers have a range of preferred policy options that would potentially reduce informal payments. The top-down or ‘traditional’ measures, such as transparency, accountability and punishment, were of relatively less importance than measures that improved earnings and career development and moved away from cash transactions. This implies that health workers prefer the opportunity for private practice and to be rewarded/incentivised for adhering to rules as they both contribute to higher earnings. It further implies that top-down measures are important but not sufficient, while bottom-up measures based on what staff perceive as important may be most effective. The finding of larger preferences for cashless transaction suggests that a cashless payment such as electronic payment (or prepayment scheme) not only reduces the transaction burden but also reduces some ‘loopholes’ of infraction. Improving remuneration and moving away from cash transactions will improve working conditions and potentially reduce the opportunity for engaging in informal payments. However, this would have important cost implication (ie, payment and monitoring costs) especially for incentive payments like salary increments ^65,66^, and would require adequate staffing to offer time off for private practice.^54^ Allowing dual practice also may need strong government regulation, since its presence might negatively affect public health provision, quality, and equity when staff struggle to balance the two sectors with respect to better earnings/benefits.^54,67^ Since Tanzania is moving towards cashless transactions in public facilities through a mandatory universal health insurance for universal health coverage,^68^ cashless payment is highly preferred.

 This study has a number of strengths. Ours is the first study to use DCE to health workers in order to inform policy interventions against informal payments and was able to examine how these varied across different subgroups of staff as a guide to policy-makers to develop targeted interventions. However, it had some limitations. First, we did not sample health workers from private facilities and lower-level facilities (dispensaries), meaning our results may not be generalisable to these settings. Second, our DCE is subject to the shortcomings of the method, such as the use restricted set of attributes and levels which affects the realism; typical focus on stated preferences as opposed actual decision-making; and challenge of not knowing to what extent the respondents will be able to easily appreciate or trust the job attributes currently not available in the job markets.^19,24^ Third, there is a possibility of reporting bias in some variable like the indicator of being engaged in informal payments previously because of being self-reported and sensitive. However, with that knowledge we placed this question towards the end of the questionnaire to enable us to build a good rapport and make respondents comfortable.^37^ Fourth, there is limited external validity of our findings (generalizability) because of our sampling technique. Our sample of providers was derived through convenience sampling based on those present at surveyed facilities on the day, and selected purposively to include individuals in different departments and levels of seniority. However, this was preferred to capture the diversity of participants in a context with limited health workforce. Future research should sample providers randomly, assess preference from non-public providers, and assess the long-term retention and satisfaction for interventions that are currently implemented.

## Conclusion

 This study highlights the importance of improving both financial and non-financial job attributes as preferred features by health workers, with the potential of addressing informal payments. The finding that preferences on incremental salary top-up, disciplinary measures at district level, working environment and opportunities for promotion/training varied across subgroups of staff echoes the need of targeted intervention packages (designing for differences) as opposed to a single strategy in an attempt to reduce informal payments. Implementing preferred multiple strategies (aligning incentives) could possibly incentivise health workers to revert their behaviours of engaging in informal payment. Thus, our findings reinforce the need to improve remuneration/earnings, working environments, and career development as these might help in reducing chances of engaging in informal payments in Tanzania.

## Acknowledgements

 The preliminary findings of this study were presented at the 16th World Congress of Public Health, held virtually, in 2020. We would like to thank all healthcare providers, health managers and all health stakeholders who participated or facilitated the planning, workshops, and fieldwork for data collection. We also thank the whole ACE health research team at IHI and LSHTM. We further acknowledge the support of research team for data collection including data collectors and field coordinators.

## Ethical issues

 Ethical approval for the research project was obtained from the Institutional Review Board of the Ifakara Health Institute (IHI/IRB/No: 009-2018), from the National institute for Medical Research (NIMR/HQ/R.8a/Vol. IX/2812) for national clearance, and from the London School of Hygiene and Tropical Medicine (LSHTM Ethics Ref: 16248). All study participants were given an information sheet explaining the project and the voluntary nature of participation, and were given a written consent form to sign before proceeding with an interview. The information sheet and consent form were both approved by the ethics committees prior to the start of the research.

## Competing interests

 Authors declare that they have no competing interests.

## Authors’ contributions

 AA, DB, MM, EH, BA, and PB designed the discreet choice experiment. PB oversaw the data collection activities. PB, BA, and AA analysed the data. PB wrote the first draft of the manuscript. AA, DB, MM, EH, BA together with PB involved in the data interpretation, presentation, and revision of the manuscript. All authors read and approved the final manuscript.

## Disclaimer

 The views presented in this publication are those of the author(s) and do not necessarily reflect the UK government’s official policies or the views of ACE or other partner organisations. For more information on ACE visit https://www.ace.soas.ac.uk.

## Funding

 This publication is an output of the ACE Research Consortium funded by UK Aid from the UK Government [Contract P07073].
